# How Does a Voltage Sensor Interact with a Lipid Bilayer? Simulations of a Potassium Channel Domain

**DOI:** 10.1016/j.str.2007.01.004

**Published:** 2007-02

**Authors:** Zara A. Sands, Mark S.P. Sansom

**Affiliations:** 1 Department of Biochemistry, University of Oxford, South Parks Road, Oxford OX1 3QU, United Kingdom

**Keywords:** CELLBIO

## Abstract

The nature of voltage sensing by voltage-activated ion channels is a key problem in membrane protein structural biology. The way in which the voltage-sensor (VS) domain interacts with its membrane environment remains unclear. In particular, the known structures of Kv channels do not readily explain how a positively charged S4 helix is able to stably span a lipid bilayer. Extended (2 × 50 ns) molecular dynamics simulations of the high-resolution structure of the isolated VS domain from the archaebacterial potassium channel KvAP, embedded in zwitterionic and in anionic lipid bilayers, have been used to explore VS/lipid interactions at atomic resolution. The simulations reveal penetration of water into the center of the VS and bilayer. Furthermore, there is significant local deformation of the lipid bilayer by interactions between lipid phosphate groups and arginine side chains of S4. As a consequence of this, the electrostatic field is “focused” across the center of the bilayer.

## Introduction

Voltage-gated potassium (Kv) channels play a key role in electrically excitable cells (Hille, 2001). Recent structural and biophysical (Swartz, 2004, Yellen, 2002) studies have focused on the mechanism of voltage sensing by these channels. Structural data suggest that the voltage sensor (VS) of Kv channels forms a largely independent domain. A high-resolution structure of an isolated VS domain from the archaebacterial channel KvAP has been determined (Jiang et al., 2003a, Jiang et al., 2003b). This structure is broadly consistent with molecular physiology and spectroscopic data and thus is likely to represent a major physiological conformation of the VS within Kv channels (Aggarwal and MacKinnon, 1996, Bezanilla, 2000, Chanda et al., 2005, Jiang et al., 2003b, Long et al., 2005b, Phillips et al., 2005, Posson et al., 2005, Ruta et al., 2005, Seoh et al., 1996, Starace and Bezanilla, 2004). Furthermore, the isolated voltage sensor of KvAP forms a stably folded domain when in either a phosphatidylcholine (PC) or a 3:1 phosphatidylcholine/phosphatidylglycerol (PC/PG) lipid bilayer environment (Cuello et al., 2005). It is thought that the VS, or elements of its structure, move in response to change in voltage across a cell membrane. Consistent with this, the VS appears capable of adopting different orientations relative to the pore domain of Kv channels (Lee et al., 2005), although it is unclear whether all of these orientations are possible for a Kv channel in situ in a membrane. The structure of the VS in a mammalian channel, Kv1.2, is similar to that of the isolated KvAP VS, and packs rather loosely against the pore domain (Long et al., 2005a, Long et al., 2005b). However, the exact nature and extent of depolarization-induced VS motion remains unresolved (Aggarwal and MacKinnon, 1996, Baker et al., 1998, Bezanilla, 2000, Cha et al., 1999, Chanda et al., 2005, Glauner et al., 1999, Jiang et al., 2003b, Larsson et al., 1996, Long et al., 2005b, Mannuzzu et al., 1996, Phillips et al., 2005, Posson et al., 2005, Ruta et al., 2005, Seoh et al., 1996, Starace and Bezanilla, 2004, Yang and Horn, 1995, Yang et al., 1996, Yusaf et al., 1996). In this context, it is crucial to understand how a VS domain interacts with the surrounding lipid bilayer.

In support of the VS domain as an independent unit, homologous domains have been found in two non-Kv proteins: a voltage-sensitive phosphatase (Ci-VSP) (Murata et al., 2005) and a voltage-sensitive proton (Hv or mVSOP) channel (Ramsey et al., 2006, Sasaki et al., 2006). As both of these proteins lack discernible K channel-like pore domains, it is reasonable to assume that the voltage-sensing domains and pore-forming domains of Kv channels can function independently.

The VS is composed of four transmembrane (TM) helices, S1–S4. The isolated VS domain of KvAP revealed that the S3 helix was distorted into two distinct helical segments, namely S3a and S3b, where the C-terminal S3b segment and the N-terminal portion of the S4 helix (the latter termed the S4a helix; Sands et al., 2005) are packed tightly together to form a helix-turn-helix motif, termed a VS paddle.

S4 is an unusual TM helix in that it contains several basic amino acid side chains which act as the primary voltage-sensing elements. This pattern of basic side chains within S4 is conserved among Kv channels and also in voltage-activated calcium and sodium channels, the voltage-sensitive phosphatase, and the Hv channel. The question arises as to how the S4 helix is accommodated within a lipid bilayer. Following the determination of the Kv1.2 crystal structure (Long et al., 2005a), it was apparent that the gating charges (i.e., Arg side chains) located along the S4 helix of the VS are likely to interact with the surrounding lipid environment. A limited number of studies have attempted to probe these interactions (Freites et al., 2005, Ruta et al., 2005, Sands et al., 2005, Schmidt et al., 2006). However, there is still considerable debate over the exact extent of these interactions, and over the exact location and lipid exposure of the Arg side chains. In particular, it remains uncertain whether the charges of S4 are completely shielded from interactions with the lipid by other regions of the VS protein, or whether some degree of local reorganization of the lipid bilayer may accommodate the voltage-sensing S4 helix. A related question is that of how the S4-containing VS domain is inserted into a bilayer. An S4 helix derived from KvAP may be biosynthetically inserted into a membrane (Hessa et al., 2005), and S4 helix peptides also may insert into a bilayer in vitro (Fernández-Vidal et al., 2006).

Molecular dynamics (MD) simulations provide a computational approach to exploring structure/function relationships of ion channels and related membrane proteins (Roux and Schulten, 2004) and the interactions of membrane proteins with their lipid bilayer environment (Deol et al., 2006). Recently, atomistic MD simulations have been used to explore the interactions of an isolated S4 helix spanning a lipid bilayer (Freites et al., 2005) and of a Kv1.2 channel with a lipid bilayer (Treptow and Tarek, 2006). However, these studies were of trajectories of duration of less than 10 ns, and so one should be cautious in their interpretation in terms of VS/lipid interactions, as simulations of ∼20 ns duration do not fully sample protein and lipid interactions (see, e.g., Deol et al., 2006). Accordingly, more extended (>20 ns) simulations are here performed in order to allow improved sampling of the lipid/protein contacts. In the current study, we perform two 50 ns duration MD simulations to investigate how the isolated VS domain of KvAP behaves in a “default” zwitterionic PC lipid bilayer and in a mixed anionic PC/PG (3:1) lipid bilayer. The latter environment mimics the lipid composition of cell membranes and has been used in experimental studies of the VS domain. The results reveal penetration of water into the center of the VS, and local deformation of the lipid bilayer by interactions of S4 side chains with phospholipid head groups. This is in agreement with the simulations of the isolated S4 helix (Freites et al., 2005) and, indeed, in terms of side-chain/lipid interactions, with recent experimental data (Schmidt et al., 2006).

## Results

### The Simulations: Progress and Stability

The conformational stability of the isolated VS in the PC and PC/PG lipid bilayer environments was measured by the degree of conformational drift of the protein from the structure used to initiate the simulations (i.e., the X-ray structure of Protein Data Bank [PDB] ID code 1ORS). This was evaluated by measuring the root-mean-square deviation (rmsd) of the Cα atoms from their starting coordinates. Each simulation shows an initial jump followed by a slower rise to a plateau of ∼1.8 Å for all Cα atoms, and of ∼1.5 Å for core fold Cα atoms (Table 1). This is comparable to the Cα rmsd values acquired for other, more “canonical,” membrane proteins in lipid bilayers, such as Aqp1 (Jensen et al., 2001). One may also compare the VS/bilayer Cα rmsd's with those of the extended (50 ns) simulation of the same domain in a decyl maltoside (DM) micelle (Sands et al., 2005) (Table 1). As anticipated, the micellar environment allows greater structural drift than a lipid bilayer environment (Bond and Sansom, 2003). This may be attributed to reduced packing constraints of the protein/detergent micelle system.

**Table 1:** Summary of Simulations

Simulation	Lipids	Waters	Cα Rmsd (Å)	Core Cα Rmsd (Å)
VS/PC	73 PC	4,066	1.8	1.4
VS/PCPG	51 PC: 16 PG	4,109	1.8	1.6
VS/micelle	94 DM	13,457	2.5	1.9

Rmsds are calculated for Cα atoms over the final 10 ns of each simulation relative to the crystal structure of PDB ID code 1ORS. Data for VS/micelle simulations are from Sands et al., (2005).

The relative flexibility of the VS in each of the lipid bilayer environments was also assessed by calculating the root-mean-square fluctuation (rmsf) of each Cα atom from its time-averaged coordinate. The overall profiles were similar, with the rmsf values being significantly higher for the interhelical regions relative to the α-helical core regions (see Figure S1 in the Supplemental Data available with this article online), as anticipated from micelle simulations (Sands et al., 2005). There is one significant difference between the two bilayer environments. The S3b-S4a region (residues ∼100–125) is more mobile in PC than in PC/PG. This suggests that the S3b-S4a region exhibits a degree of flexibility that can be attenuated by its lipid environment. Detailed analysis reveals that the motion of the S3b-S4a region during the VS/PC simulation appears to be correlated to the motion of the midsection of S3 and that this correlated motion is conserved, albeit to a lesser extent, in the VS/PCPG simulation (see Figure S2). This is the only significant correlated motion that is conserved between the VS/PC and VS/PCPG systems, and thus it may constitute a physiologically important motion that underlies voltage sensing (i.e., the S3b-S4a region of the “paddle” may move as a rigid unit relative to the rest of the VS). In this context, is it interesting that the two different structures of the intact KvAP molecule (PDB ID codes 1ORQ and 2A0L) show that the S3b-S4a region of the paddle exists in different orientations with respect to other components of the VS. It is also interesting to note that the motions of the two helical segments (S3a and S3b) which comprise the S3 helix do not appear to be significantly correlated in either the VS/PC or VS/PCPG simulations (see Figure S2).

Overall, one may conclude that the VS domain protein is conformationally stable in a bilayer environment simulation on a 50 ns timescale. Thus, it behaves like a stable canonical membrane protein, even though it is an isolated domain containing a positively charged S4 helix. Perhaps the stability of the isolated domain is not surprising, given that the two-state model of membrane protein folding (Popot and Engelman, 2000) implies that isolated TM helices and domains should be stable. However, initially one might consider it surprising that there does not appear to be a need for the exposed S4 helix to interact with other protein regions to remove the positive charges from the hydrophobic membrane environment. The way in which the lipid bilayer adjusts to the S4 helix and the possible functional consequences of this will now be explored in more detail.

### Voltage Sensor/Lipid Interactions

The nature of the interactions between the VS (especially S4) and the lipid head groups is of particular interest. Simulation studies of an isolated S4 helix (Freites et al., 2005) and of the intact Kv1.2 channel (Treptow and Tarek, 2006) inserted into a lipid bilayer have been reported and suggest a degree of distortion of the bilayer. However, the simulations were relatively brief compared to the time taken for lipids to “relax” around a membrane protein. It has been suggested that there may be a considerable energetic barrier to inserting a single arginine residue within the core of a lipid bilayer (Dorairaj and Allen, 2006), and thus it is important to examine the nature of S4/lipid interactions in the more extensively sampled simulations presented here.

**Figure 1:**
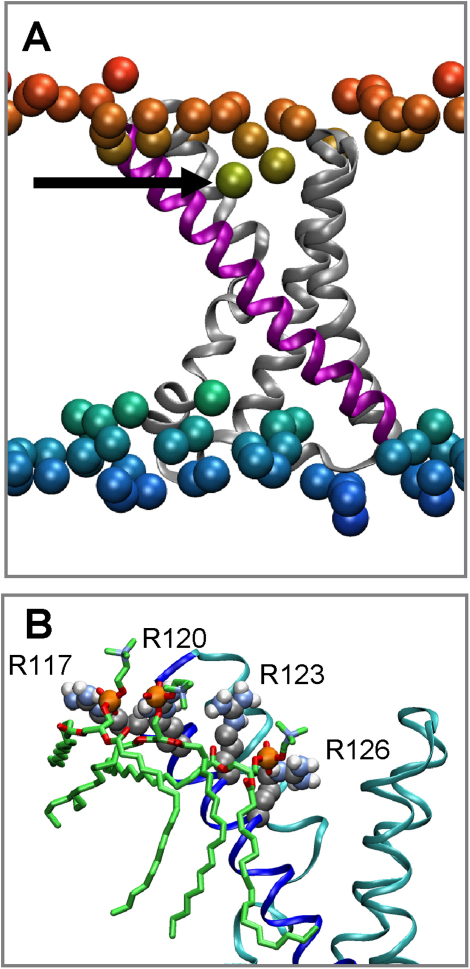
The KvAP VS in a Phospholipid Bilayer (A) The VS domain (represented in a ribbon format) at the end of a 50 ns simulation in a PC lipid bilayer. The S4 helix is highlighted in magenta and the remaining S1–S3 helices are depicted in gray. The phosphorus atoms of the lipid molecules (shown as spheres) are colored according to the magnitude of their *average* z coordinate, ranging from red at the extracellular interface to blue at the intracellular interface. The arrow indicates the P atom of a lipid molecule whose head group has been “pulled” in toward the interior of the membrane. (B) Interactions between S4 helix arginine side chains and the lipid phosphate groups from a snapshot of the VS/PC simulation at 50 ns.

As a first step, we have combined visualization of simulations with analysis of the location (along the bilayer normal) of the P atoms of the phospholipid head group (Figure 1A). Examination of snapshots from the simulations suggests that the lipids interact strongly and are “drawn” into charge filled crevices within the VS domain. Further examination indicates that the presence of the VS significantly perturbs the (local) organization of the lipid bilayer. The lipid head groups are drawn into the VS over the course of each simulation; this phenomenon effectively compresses the lipid bilayer within the immediate vicinity of the VS and results in unusual conformations being adopted by nearby lipids (see Figure S3). For instance, we observe varying degrees of lipid tail interdigitation. We also see evidence of lipid tail groups “snaking” around the hydrophobic surface of the VS. Visualization of snapshots suggests close interactions of proximal lipid phosphates with Arg side chains of S4 (see Figure 1B).

**Figure 2:**
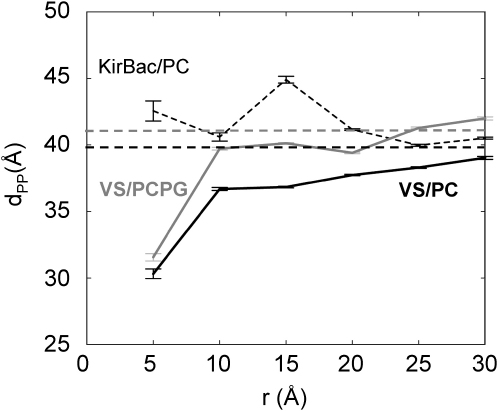
Local Deformation of the Lipid Bilayer This is measured as the average distance between upper and lower P atoms (*d_PP_*) versus the distance of the atoms in the xy plane from the center of mass of the corresponding protein (*r*) over the last 10 ns of each simulation. The *d_PP_* versus *r* curves are given for the VS/PC (black, solid line) and VS/PCPG (gray) simulations. As a control, the *d_PP_* versus *r* curve is given for a simulation of KirBac3.1 in a PC bilayer (black, broken line). The horizontal lines give the corresponding *d_PP_* values for two control PC (black dashed) and PC/PG (gray dashed) bilayer simulations in the absence of protein.

This drawing in of the lipid by the charged residues of the VS may be quantified by measuring the bilayer P-P distance as a function of radial (i.e., in the bilayer plane) distance out from the center of the protein (see Figure 2). It is evident that there is local deformation of the lipid bilayer by the VS domain. Thus, in both simulations, the P-P thickness close to the center of the protein is ∼7 Å less than that for more distant regions of the bilayer (and in control, i.e., lipid bilayer only, simulations). This is in stark contrast with other, more canonical, membrane proteins, such as the inwardly rectifying K^+^ channel KirBac3.1, which when simulated for 20 ns in a PC bilayer does not lead to localized lipid bilayer compression (A. Grottesi and M.S.P.S., unpublished data; see Figure 2).

### H-Bonding Interactions

**Figure 3:**
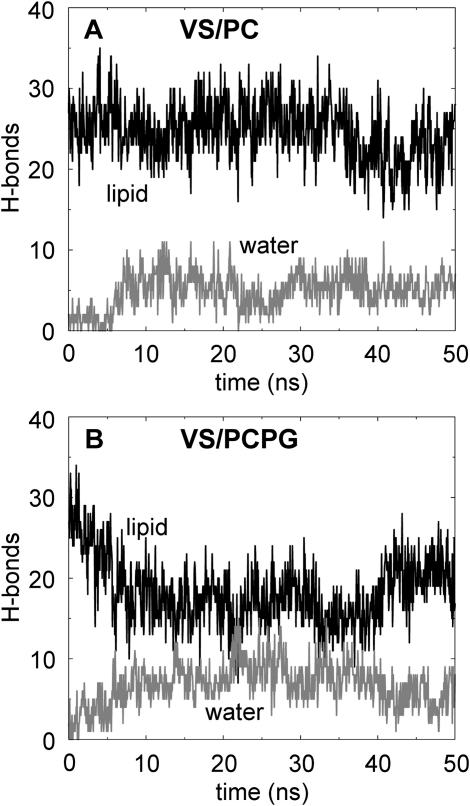
Hydrogen Bonds Formed by the S4 Helix Number of H bonds formed by the side chains of the charged residues of S4 as a function of time for simulations (A) VS/PC and (B) VS/PCPG. In each case, the numbers of H bonds with lipids and with waters are shown in black and gray, respectively.

Although the charged S4 helix is significantly (∼50%) exposed to the lipid bilayer environment during the course of each simulation, the VS domains remain conformationally stable. It is of some interest, therefore, how these gating charges are accommodated over the course of each simulation (Figure 1B). It was found that the charged residues located along S4 can form H-bond interactions with water molecules and lipid head groups (Figure 3). The total number of hydrogen bonds was monitored over the course of each simulation and it was found that when the VS was simulated in a PC environment, the S4 helix could form a greater number of H-bond contacts with its environment (lipids and waters) than if simulated in a PC/PG bilayer.

**Figure 4:**
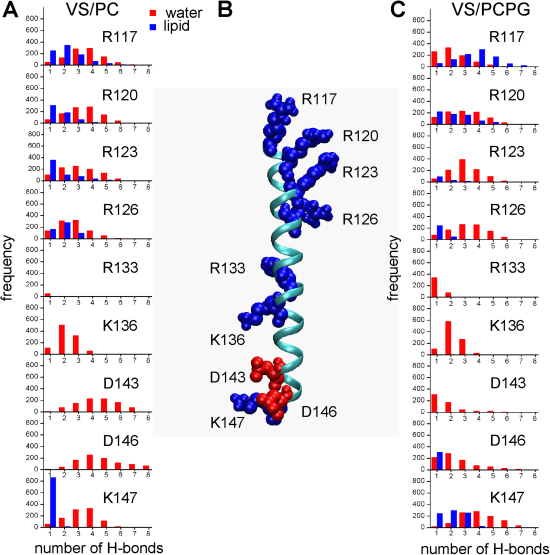
Interactions of the S4 Helix with Lipid and Water Molecules (A and C) Frequency (over the 50 ns simulation) distributions of the number of H bonds formed with the charged residues of S4. Distributions are shown for the numbers of H bonds each residue forms with water (red) and with lipid (blue) for the VS/PC (A) and VS/PCPG (C) simulations. (B) The S4 helix (represented as a cyan ribbon) taken from the start of the VS/PCPG simulation. The charged residues are depicted in space-filling format, with cationic and anionic side chains colored blue and red, respectively.

Further analysis also reveals that the lipid head groups in the mixed PC/PG have the propensity to form a greater number of H bonds with the positively charged residues located along S4 than do the lipid head groups of the PC simulation (Figure 4). This finding is hardly surprising, given that the PG head group has a greater number of potential H-bond donors and H-bond acceptors.

Each of the charged residues located along the S4 helix is involved in forming H bonds with water molecules over the course of the simulations, indicating that individual water molecules extensively penetrate the VS. However, in contrast to the gating charge arginines, the centrally located Arg133 forms a limited number of H bonds with waters. It can also be observed that the lipid head groups can form H bonds with all of the gating charge residues located toward the N terminus of S4 (Arg117, Arg120, Arg123, and Arg126) and the two charged residues located toward the C terminus of S4 (Asp146 and Lys147). Such H-bonding interactions may help stabilize the VS and the highly charged S4 helix within a bilayer environment.

### Water Penetration

**Figure 5:**
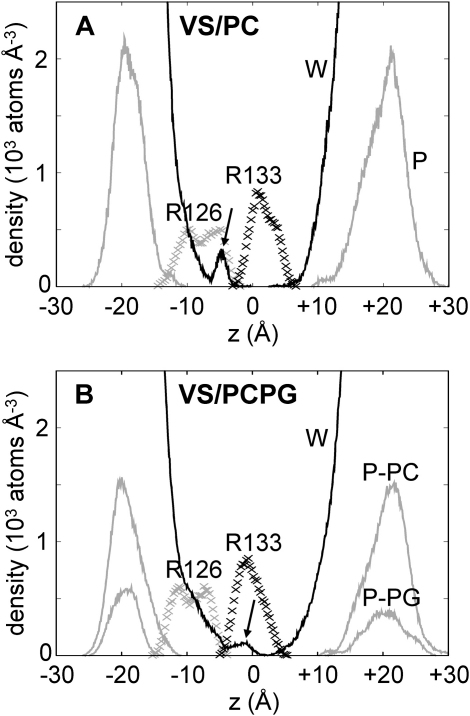
Density Profiles along the Bilayer Normal Partial densities for lipids (P atoms of head groups, gray solid lines), water (w, black solid lines), Arg126 (R126, gray crossprofile), and Arg133 (R133, black crossprofile) for simulations (A) VS/PC and (B) VS/PCPG. In the latter, the density profiles for P atoms of the PC and PG molecules are shown separately. The arrows indicate the region in the water profiles corresponding to the penetration of water toward the center of the VS domain.

Inspection of the average partial density plots for each simulation reveals a reduction in water density toward the center of the S4 helix and the marked absence of water density at a central constriction point (Figure 5). Interestingly, we note the presence of a “bump” in each of the water density profiles. In the VS/PC simulation, the increase in water density occurs at z ∼ −5 Å, which overlaps with the density of Arg126; it is therefore likely that H-bond interactions draw waters in toward this residue. For the VS/PCPG simulation, there also appears to be an overlap between the water and the Arg126 density profiles in addition to this, though there is an overlap that occurs at z ∼ −3 Å between the water and the Asp62/Arg133 salt-bridging residues, which is most likely due to the formation of H bonds between these residues and water (see Figure 4).

The partial density plots also reveal an asymmetric distribution of the lipid head groups, with the phosphorus atoms sitting lower in the lipid leaflet accommodating the gating charges. This location bias suggests that the gating charges form more favorable interactions with lipid head groups than do the charged residues located toward the C terminus of S4.

### Functional Consequences: Water, “Pore,” and Electrostatic Potentials

**Figure 6:**
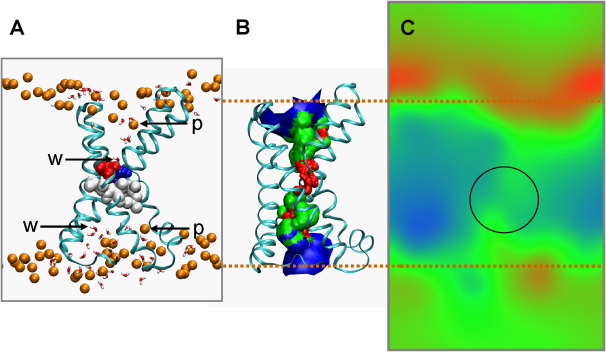
Water Penetration into the VS Domain (A) Snapshot taken at *t* = 50 ns from the VS domain in a PC bilayer simulation revealing the protein backbone (Cα trace, cyan), lipid phosphorus atoms (p, orange spheres), water molecules (w, red/white spheres) within 2.5 Å of the protein atoms, the residues Asp62 and Arg133 (red and blue, respectively) which form a salt bridge between S2 and S4, and the hydrophobic residues (Leu65, Val66, Ala96, and Leu97, shown in white) that effectively occlude the pore to the permeation of water and ions. (B) The average pore radius lining surface (red/green/blue) over the last 5 ns of the VS/PC simulation calculated using HOLE (Smart et al., 1996), along with the VS protein Cα trace (cyan) taken at *t* = 45 ns. (C) Electrostatic potential distribution in a region centered on the VS. The map is taken from a slice in the xz plane of the VS/PC system (see Experimental Procedures). The potential is shown on an RGB scale spanning ± 120 mV. The potential is focused over a highly circumscribed region about the Asp62-Arg133 salt bridge.

Analysis of the radius profile along the VS crevice reveals that there is a central constriction which (when averaged over the course of each simulation) is significantly narrower than the radius of a water molecule (see Figure 6B). The constriction stretches over a region in excess of 7 Å in length and centers on a salt bridge formed between Asp62/Arg133 and the hydrophobic residues located “below” this bond (Leu65, Val66, Ala96, and Leu97). The salt bridge effectively brings these hydrophobic residues into close proximity, and may explain why the charged residues located toward the C terminus of S4 form fewer interactions with the hydrophilic lipid head groups.

Electrostatic potential calculations (performed using the PMEPOT plug-in for visual molecular dynamics [VMD]; see Experimental Procedures) suggest that the electrostatic potential is focused across the hydrophobic residues that are brought into close proximity by the maintenance of the Asp62 and Arg133 salt bridge (Figure 6C). This is discussed further below in the context of implications for the voltage-gating mechanism of Kv channels.

### Revisiting Lipid/S4 Interactions

**Figure 7:**
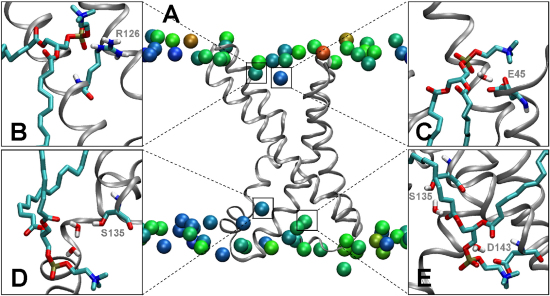
Phospholipid Molecules “Bound” to the VS (A) Average lipid and protein coordinates calculated over the last 10 ns of the VS/PC bilayer simulation. The phosphorus atoms (shown in space-filling format) of the PC bilayer are colored according to their mobility (measured as rmsf in the xy plane) of each atom; the color scale extends from blue = 0 Å^2^ to red = 48 Å^2^. (B–E) Four lipid molecules whose average z value coordinate has undergone the largest displacement from the initial lipid bilayer setup are shown in further detail, along with residues to which they form H-bonding interactions at *t* = 50 ns.

The lateral mobility of each lipid (head group) was assessed by calculating the mean-square fluctuation (msf) of the lipid phosphorus atoms over the last 10 ns of each simulation (Figure 7). In general, the slower lipids were found to be in closer proximity to the VS, although discrete clusters of slow lipids were observed in the “bulk” environment. Such slow islands have been observed in other protein-lipid simulations (e.g., the bacterial transporter BtuB; Pikunic, J., Khalid, S., and M.S.P.S., unpublished data) and seem to correlate with lipid-binding surface motifs on the protein.

Visual inspection of the equilibrated portion of each trajectory revealed that some of the lipid head group H-bonding interactions to the VS were facilitated by interchanging water molecules. It was also clear that some of the more mobile lipids could maintain continual H-bonding interactions with the VS throughout the simulation period. This is best understood by considering the lipid head group that binds to R123 and over time migrates down to R126 (Figure 7B).

## Discussion

Our analysis has examined the interactions of the isolated VS domain of KvAP in lipid bilayer environments. These simulations, conducted over a timescale that allows reasonable sampling of lipid/protein interactions, reveal how it is that the isolated VS domain of KvAP can form a stably folded domain in either a PC or a mixed PC/PG lipid bilayer environment. This provides further support for the contention that the VS domain can subsist independently of the pore-forming domain of Kv channels.

The VS domain and, moreover, the charged S4 helix, appear to be accommodated in both a PC and a PC/PG bilayer through substantial H-bonding interactions of charged residues with water molecules and lipid head groups. These favorable H-bonding interactions lead to two phenomena occurring, namely the extensive penetration of water molecules into the VS and the significant local perturbation of the lipid species within close proximity of the VS. This latter process is the consequence of favorable H bonding between lipid head groups and charged residues situated in the crevices of the VS. Such interactions effectively draw the coordinated lipid species into the VS and effectively reduce the bilayer width (∼7 Å over 50 ns) within the immediate vicinity of the VS domain.

Lateral lipid mobility analysis revealed another interesting phenomena occurring in the lipid bilayer: discrete clusters of slow lipids within the immediate vicinity of the VS and in the bulk lipid bilayer environment. Favorable nonbonding interactions between the VS and the lipids (head groups/tails) may account for the decreased lateral mobility of the lipid species that comprise these slow islands.

Thus, these simulations reveal that the lipid bilayer does not simply function as an unstructured fluid into which the protein is embedded; indeed, they reveal a picture of a structured yet dynamical lipid bilayer that can reorganize to accommodate the (charged) protein. A similar situation was seen in simulations of the isolated S4 helix in a bilayer (Freites et al., 2005). Furthermore, recent experimental studies (published since the initial version of this paper was submitted) strongly support the existence of interactions between the arginine side chains of the voltage-sensor domain of KvAP and the phosphates of the lipid head groups (Schmidt et al., 2006).

These simulations also reveal that the S3b-S4a region is significantly more mobile in PC than in PC/PG. This suggests that the S3b-S4a region exhibits a degree of flexibility that can be attenuated by its environment. The sensitivity of the S3b-S4a region to its environment may account for the different orientations of the S4 helix observed in the Kv channel structures elucidated thus far (Jiang et al., 2003a, Lee et al., 2005, Long et al., 2005a).

Another important finding from these simulations is that there is a central constriction in the apparent VS cavity which is too narrow to facilitate the passage of water molecules through the VS. The constriction is centered on a salt bridge formed between Asp62 and Arg133 and the hydrophobic band of residues that sit beneath this bond (located toward the N terminus of S1, C terminus of S2, C terminus of S3a, and C terminus of S4). Electrostatic potential calculations reveal that the lipid bilayer potential is focused across this highly circumscribed hydrophobic region. Given that the isolated VS of KvAP is broadly consistent with a range of biophysical and physiological data for an open state of the channel, it is possible that this salt bridge and the hydrophobic occlusion it maintains may be where the voltage is focused in the open conformation under physiological conditions.

It should be noted that the simulations described here were performed on the high-resolution (1.9 Å) structure of the isolated VS domain from KvAP (PDB ID code 1ORS). However, it should also be noted that the conformation of this domain is different in the two structures of the intact KvAP channel (PDB ID codes 1ORQ and 2A0L). This may reflect a degree of distortion (or adoption of a “minor” conformation) of the KvAP channel conformation in the absence of a lipid bilayer environment. The isolated VS domain structure is rather closer in conformation to that of the VS domain in the Kv1.2 structure (PDB ID code 2A79; see, e.g., Figure 4 in Sands et al., 2005 for a comparison). However, there are still some differences between the KvAP VS and the Kv1.2 VS. In particular, the salt-bridge interaction between R133 and D62 which helps to maintain the hydrophobic plug inside the KvAP VS may be absent from the VS of Kv1.2. However, given the lower resolution (2.9 Å) of the Kv1.2 structure (reflected in the absence from the coordinate set of side chains for helices S1 and S3, and for any atoms of the S1/S2, S2/S3, and S3/S4 loops), a detailed comparison may have to await a higher-resolution structure (or model) for the VS of Kv1.2.

Overall, we may conclude that the lipid environment can accommodate the highly charged S4 helix of the VS through extensive H-bonding interactions and that lipid interactions may modulate the behavior of the VS (as seen in recent experimental studies; Schmidt et al., 2006). It is also likely that in situ the voltage is focused across the narrow hydrophobic plug maintained by an Asp62-Arg133 salt bridge and that the exposure of the gating charges from the extracellular to the intracellular aspect of the cell would require disruption of this S2-S4 salt bridge. The compression of the lipid bilayer in the vicinity of the VS would effectively reduce the distance the gating charges would have to travel if voltage gating occurred via a large-scale motion as postulated by the paddle model (Jiang et al., 2003b, Long et al., 2005b, Ruta et al., 2005). It is also likely that any movements of the gating charges may be facilitated by coordinating lipid species, which would effectively reduce the energy barrier to gating charge translocation. Therefore, the paddle model of the voltage-sensing mechanism should not be ruled out on energetic considerations.

It is interesting to consider that our overall conclusions concerning the distortion of a lipid bilayer by insertion of the VS domain are very similar to those reached by Freites et al. (2005) on the basis of their simulations of the isolated S4 helix and more recently of the VS domain (Freites et al., 2006). This suggests a certain robustness of the underlying result to whether a single S4 helix or a more complex S4-containing domain is used. Perhaps this is indicative of an early stage in evolution where a more complex domain “captured” a putative voltage-sensing helix. It would be interesting in this context to examine in detail the behavior of more distant homologs of the Kv channel VS domain (Murata et al., 2005, Ramsey et al., 2006, Sasaki et al., 2006).

## Experimental Procedures

### Protein Model

The initial voltage-sensing domain model (residues 19–147) was taken from the 1.9 Å isolated voltage-sensor crystal structure (PDB ID code 1ORS) (Jiang et al., 2003a). Side-chain ionization states were determined based on pK_A_ calculations performed using the program WHAT IF (Vriend, 1990) combined with locally written code. From these calculations, it was predicted that all of the ionizable side chains would be present in their default (at pH 7) ionization states with the exception of D146, which was considered to have an equal propensity for adopting either a neutral or protonated state. A study of the KvAP VS in a DM detergent micelle revealed that simulations where D146 is present in either a neutral or protonated state yield remarkably similar results (Sands et al., 2005). Thus, the simulations of the KvAP VS in PC and PC/PG were performed with all of the ionizable residues in their default states (at pH 7), including D146.

### Setup of Bilayer Systems

An equilibrated PC bilayer comprised of 128 lipid molecules (64 lipid molecules per leaflet) was obtained, courtesy of D.P. Tieleman (http://moose.bio.ucalgary.ca) for the VS/PC simulations. To set up the 3:1 PC/PG bilayer, lipids of the PC bilayer were “mutated” into PG species. To mutate a PC into PG, the choline group needs to be replaced with a glycerol group. A glycerol group already exists in PC which connects the phosphate group to the fatty chains. For each choline group, the rmsd of all possible choline/glycerol pairs in the bilayer was calculated after least-square-fitted superimposition. The choline group of each lipid was replaced by the glycerol group of the pair with the lowest rmsd. This ensured that the final bilayer was as close to an equilibrated state as possible.

As the PG lipid species carries a net negative charge, it was necessary to establish a symmetrical PC/PG bilayer while maintaining an even distribution of the two lipid species if the integrity of the membrane were to be maintained over the course of an MD simulation. The random selection of lipids for mutation could not guarantee these requirements. Instead, the positions for PG lipids were manually chosen to avoid PG clusters. To achieve this, each leaflet of the PC bilayer was divided into an 8 × 8 grid, with one PC lipid occupying each grid point. On each row and column, one PC lipid out of every four was mutated into PG. The VS was inserted into the resultant PC and PC/PG bilayers and overlapping lipid molecules were removed.

Each protein/bilayer system was energy minimized and then solvated by superimposition of a box of preequilibrated single point charge water molecules, followed by removal of any water molecules too close to either protein or lipid molecules. The resultant systems were then subjected to further energy minimization. Potassium and chloride ions were added to each system by random replacement of water molecules, until a final concentration of ∼0.1 M was achieved. The resultant systems were subjected to a further stage of energy minimization. Prior to the production run, a 1 ns equilibration run was performed during which all of the heavy (i.e., non-H) protein atoms were harmonically restrained with an isotropic force constant of 1000 kJ mol^−1^ nm^−1^. Restrained MD runs were performed at 300K for each protein/bilayer system. Finally, all positional restraints were removed and production run simulations were performed for each system.

### Simulation Details

Simulations were performed using GROMACS 3.1 (Lindahl et al., 2001) (see http://www.gromacs.org). The GROMOS96 force field was used for both simulations (Scott et al., 1999). The parameters for PC and PG were based on GROMOS96, supplemented with additional bond, angle, and dihedral terms.

All energy minimization procedures used <1000 steps of steepest descent method, in order to relax any steric conflicts generated during system setup. Long-range electrostatic interactions were calculated using the particle mesh Ewald (PME) method, with a 10 Å cutoff for the real space calculation (Darden et al., 1993). A cutoff of 10 Å was used for the van der Waals interactions. The simulations were performed at constant temperature, pressure, and number of particles. The temperatures of the protein, lipid, and solvent (waters and ions) were separately coupled using the Nose-Hoover thermostat (Nose, 1984) at 300K, with a coupling constant τ_T_ = 0.1 ps. System pressures were semi-isotropically coupled using the Parrinello-Rahman barostat (Parrinello and Rahman, 1981) at 1 bar with a coupling constant τ_P_ = 1 ps and compressibility = 4.5 × 10^−5^ bar^−1^. The LINCS algorithm (Hess et al., 1997) was used throughout to constrain bond lengths. The time step for integration in both simulations was 2 fs, and the coordinates and velocities were saved every 5 ps. Simulations were performed on a 68 node PC cluster with dual Xeon4 processors. All analyses used GROMACS and/or locally written code. Electrostatic potential maps were obtained from the average of a PME calculation performed over 100 configurations spanning the last 10 ns of each simulation using the PMEPOT plug-in for VMD (Aksimentiev and Schulten, 2005, Humphrey et al., 1996).

## Supplemental Data

Document S1. Three Figures
