# Supplementary material for: How Does a Voltage Sensor Interact with a Lipid Bilayer? Simulations of a Potassium Channel Domain
Source: Structure. 2007 Feb;15(2):235–44. doi: 10.1016/j.str.2007.01.004 (PMC1885962; doi:10.1016/j.str.2007.01.004)
Supplement: Document S1. Three Figures [file mmc1.pdf]

**Structure *15***

## Supplemental Data

# How Does a Voltage Sensor Interact with a Lipid Bilayer? Simulations of a Potassium Channel Domain

**Zara A. Sands and Mark S.P. Sansom**

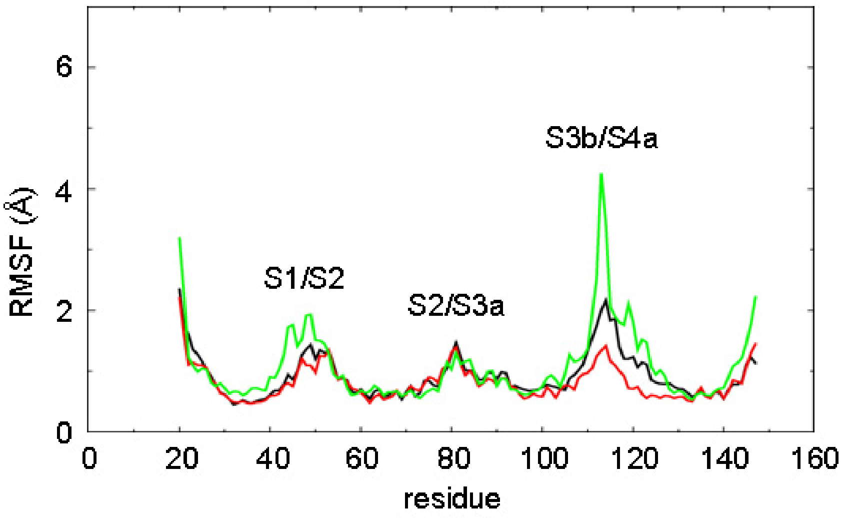


Figure S1. Conformational Flexibility Measured as Root-Mean-Square Fluctuation (RMSF) of the Cα Atoms from the Initial (1ORS) Structure

The Cα RMSFs over 50 ns are shown for simulations: VS/PC (black), VS/PCPG (red) and VS/DM (green).

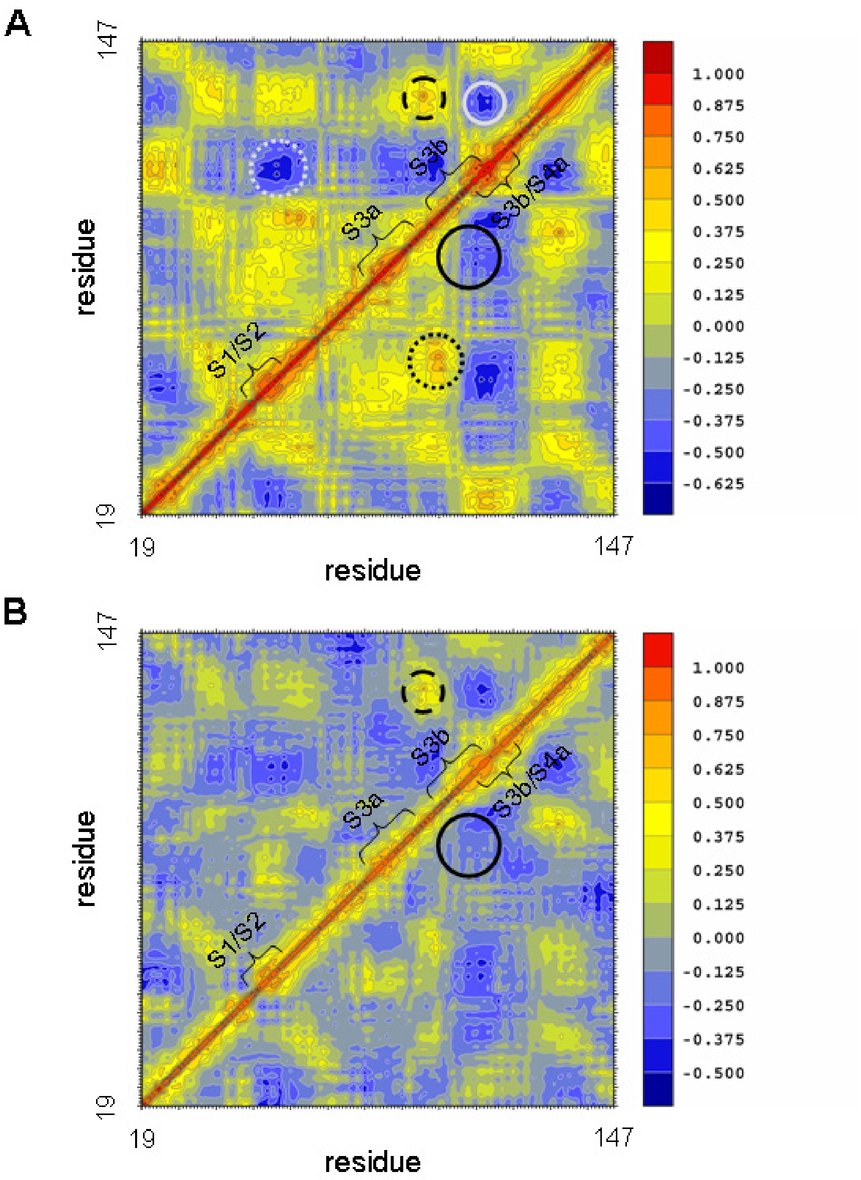


Figure S2. Correlated Motion Matrices Calculated from *t* = 7 ns to 50ns, Revealing Correlated (Red) and Anticorrelated Motions of the VS Over the Course of the **A** VS/PC and **B** VS/PCPG Simulations

The correlated motions between S3a/S3b, mid-section of S3/S4a, S3b/S4a, N terminus of S2/mid-section of S3 and N-terminus of S2/S3b-S4a are shown as solid black, black dash, solid grey, black dotted and grey dotted circles respectively.

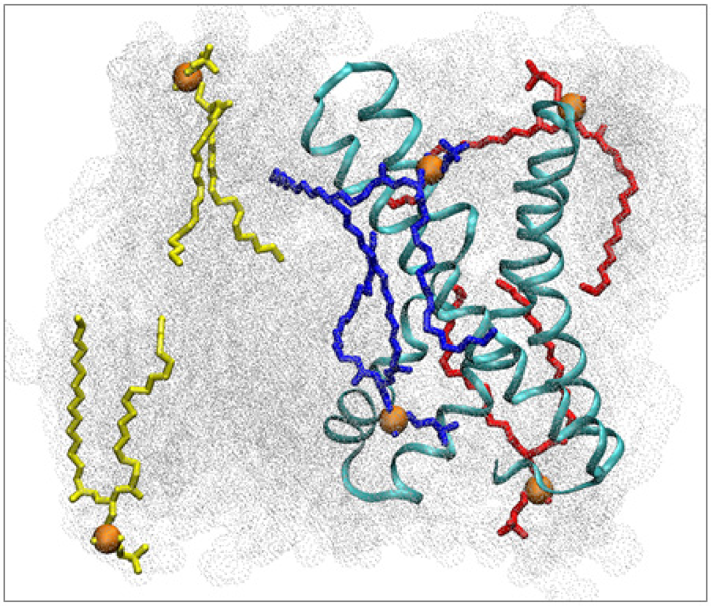

Figure S3. Snapshot of VS (Cyan) Taken from VS/PC Simulation at $t$ = 50 ns

The lipid bilayer is shown (grey) along with two lipids that are: towards the edge of the simulation box (yellow), in close proximity to the S1-S2 helices (red), and in close proximity to the S3-S4 helices (blue). The lipid phosphorous atoms are also depicted for clarity (orange).
